# A case report of simultaneous orthotopic liver transplantation and jejunectomy

**DOI:** 10.1186/s12893-016-0184-8

**Published:** 2016-09-22

**Authors:** Guoyong Chen, Sidong Wei, Zhongwu Zou, Jianjun Sun, Gaofeng Tang, Jianbin Chen, Shaotang Zhou

**Affiliations:** 1 Center of Hepatopancreaticobiliary Surgery, Henan Provincial People’s Hospital, Zhengzhou, Henan 450003 China; 2 Hubei Vocational-Technical College School of Medicine, Xiaogan, 432000 China; 3 Center of Hepatopancreaticobiliary Surgery and Liver transplantation, 100 Xisihuang Middle Road, Fengtai District, Beijing, 100039 China

**Keywords:** Acute-on-chronic, End-stage liver disease, Portal vein thrombosis, Liver transplantation, Phlebothrombectomy, Jejunectomy

## Abstract

**Background:**

Liver transplantation (LT) accompanied by jejunectomy to treat patients with acute or chronic hepatic cirrhosis with thrombosis in the portal system is extremely rare.

**Case presentation:**

A 47-year-old man presented with hematemesis and melena, and a diagnosis of decompensated cirrhosis, chronic portal vein thrombosis (PVT) and secondary gastro-esophageal variceal hemorrhage was made. Coagulants were administered, but portal vein thrombi occurred rapidly, and gastrointestinal bleeding recurred shortly thereafter. The patient underwent LT, phlebothrombectomy and a partial jejunectomy. His recovery from a fistula was uneventful, and follow-up visits over 70 months were unremarkable.

**Conclusion:**

Liver transplantation and partial jejunectomy is a feasible and effective surgical option for select patients with end-stage liver disease accompanied by acute portal venous thrombosis.

## Background

Chronic portal vein thrombosis (PVT) is a vascular disease that commonly occurs in end-stage liver disease patients waiting for liver transplantation (LT), and it causes challenges for transplant specialists. Nearly three decades ago, advanced cirrhosis and PVT were contraindications for liver transplantation because of surgical technique limitations. In 1985, Shaw et al. first reported using a venous graft conduit to bypass the thrombotic segmental vessel to successfully overcome surgical limitations during LT [1].

More clinical cases were subsequently reported about this entity because of imaging technology progress, such as ultrasound, computerized tomography and magnetic resonance that can detect disease alone or in combination before transplantation [2]. Its prevalence in orthotopic liver transplantation candidates ranged from 2.1 to 21 % [3–5].

Thrombi in the main portal vein can extend to different splanchnic veins because of predisposing factors. Based on how rapidly PVT and collateral veins develop, PVT was clinically classified as either acute or chronic [6–8]. If there is a lack of collateral veins, thrombosis develops quickly and results in congestive ischemia and subsequent gut necrosis, which are typical clinical presentations of acute PVT. However, chronic PVT, which is more common, is almost asymptomatic and develops over months or years. Yerdle et al. graded 4 forms of chronic PVT based on the extent of occlusion and mesenteric veins involved [9], and many surgical strategies had been subsequently developed to revascularize the maintain blood flow to the transplanted liver [10–12]. There have been no cases of LT candidates with acute PVT that is accompanied by intestinal necrosis. Here, we present the first case of a patient with acute-on-chronic PVT with intestinal necrosis that occurred during LT that was used to treat end-stage liver cirrhosis.

## Case presentation

A 47-year-old male patient was admitted with esophagogastric hemorrhage secondary to portal hypertension derived from decompensated cirrhosis. At the initial consultation, his chief complaint was hematemesis and two episodes of melena. His past medical history was viral hepatitis B with HBsAg + and HBcAb + for more than 10 years, and his surgical history included splenectomy and periesophagogastric devascularization for portal hypertension, hypersplenism and splenomegaly, and cholecystectomy for gallstones one year before, at a different hospital. His vital signs were as follows: temperature, 36.7 °C; blood pressure, 120/68 mmHg; respiratory rate, 20; and heart rate, 84. He had scleral icterus in both eyes. Physical examination showed negative chest and cardiac findings. Tenderness and rebound pain was marked, and shift dullness was remarkable. His blood work results were as follows: white blood cell count, 16.6 × 10^9^/l; red blood cell count, 2.47 × 10^12^/l; platelet cell count, 183 × 10^9^/l; and hemoglobin, 67 g/l. Stool hemoccult test results were positive (+++). His liver function profile was as follows: total bilirubin, 84 μmol/l; ALT, 319; AST, 347; albumin, 32.3 g⁄l; C-reactive protein, 107 mg/l; alpha-fetoprotein, normal; carcinoembryonic antigen concentration test, normal; and amylase, unremarkable. The patient’s coagulopathy parameters were as follows: PT, 19.3 s; PT%, 43.6 %; fibrinogen, 1.4 g/l; APTT, 120.9 s; and TT, 21.3 s.

**Fig. 1:**
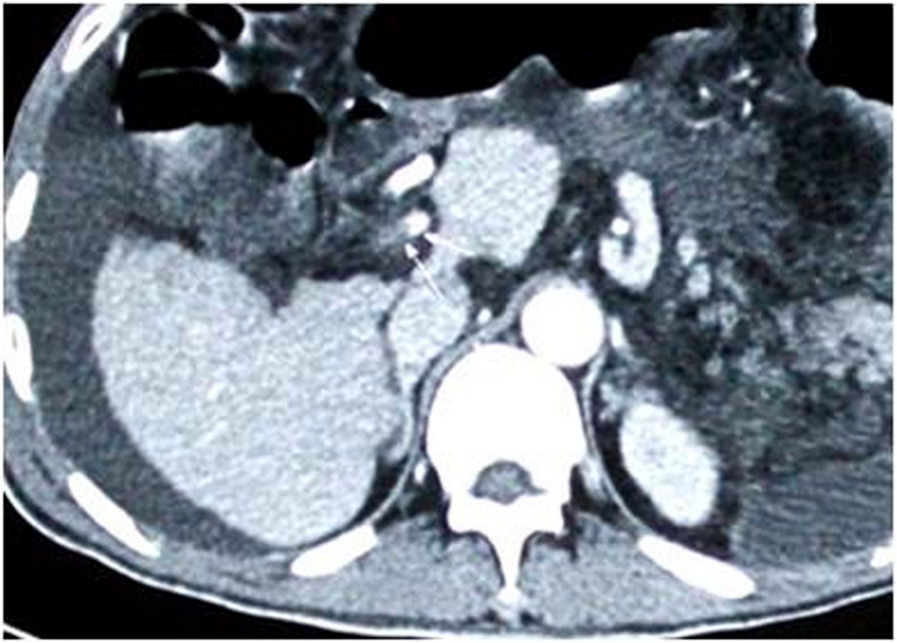
Computerized tomography scan showed ascites, cirrhosis, emboli in portal vein system (arrows)

Paracentesis fluid revealed serosanguinous ascites, and bacterial culture showed that no bacteria were present. Enhanced computerized tomography results showed hepatic nodular regeneration, moderate ascites, a dilated bowel, and emboli in the portal vein and the superior mesenteric vein that extended to the distal and tributary veins (Fig. 1). Melena and hematemesis reoccurred after administering coagulants including thrombase and tranexamic acid, which were administered for a few days. The patient was moved to transplant surgery to await a donated liver.

**Fig. 2:**
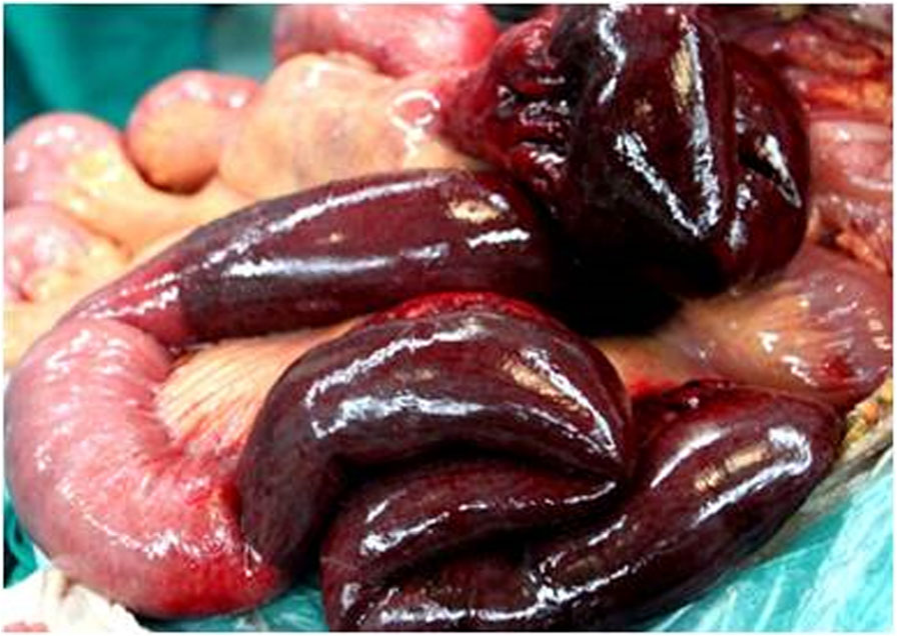
Appearance of the to-be excised jejunum

**Fig. 3:**
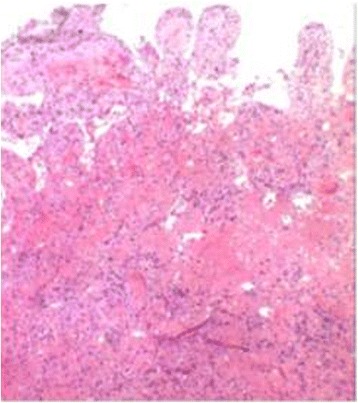
H&Estaining showed necrosis in the intestinal wall

Two days later, the liver from a 35-year-old deceased male with ABO-compatibility was available, and liver transplantation and thrombectomy were planned. After entry into the abdominal cavity, approximately 2.5 l of ascites was removed using suction. The abdominal cavity was explored; the liver had several nodules, the spleen and gall bladder had been excised, complete occlusion of the portal vein and the superior mesenteric vein (Grade III-IV) was found, and two segments of the proximal jejunum were purplish-black in color. A short distal vein arcade was fully occluded, and there was intestinal necrosis with intestinal tone and no perforation (Figs. 2 and 3). Ascites culture results were negative, which suggested that there was no bacterial translocation. Intra-operative ultrasound confirmed the permeability of the superior mesenteric artery, which had a palpable pulse. Further histology results showed nodular hyperplasia that excluded small cancer. Orthotopic LT was then performed using a piggy-back caval anastomosis, and portal vein eversion was then conducted to remove the thrombi in the portal and superior mesenteric veins. These veins were cavernous, with numerous small thrombi that were flushed out via blood flow. Portal venous blood flow was restored and confirmed intra-operatively using a Doppler ultrasonic flow meter (data not shown), and 25 units of plasma and packed red cells were transfused. Approximately 100 cm of gangrenous jejunum was subsequently resected from Treitz’s ligament, and an end-to-end anastomosis of the jejunum was performed to maintain intestinal continuity. The patient recovered fully, although a fistula occurred, which resolved upon draining.

Medications such as the following were administered: immunosuppressive therapy to treat immune rejection, potent broad-spectrum antibiotics to treat potential infection, anti-coagulant agents to treat re-embolism, and lamivudine and immunoglobulin to prevent hepatitis B viral infection recurrence. The allograft functioned well, and ALT, AST and bilirubin levels decreased daily, while albumin levels increased. Thirteen days after OLT, a fistula occurred in the anastomosis, and laparoscopic surgery was performed again to allow suction and access to the abdominal cavity. A 4-cm mushroom-shaped tube was inserted into the near jejunum to allow drainage, and a feeding tube was also inserted into the distal intestine. After about 3 weeks, the patient was recovering well. Seventy months after surgery, the patient’s allograft was functioning well and his medical history has been uneventful.

## Discussion

We report the case of a cirrhosis patient with acute-on-chronic PVT. Chronic PVT is a commonly encountered problem in LT candidates [13], and it is characterized by a slow progression into cavernomatosis transformation and portal hypertension. Refractory ascites, gastrointestinal bleeding and disproportional abdominal pain are the physical findings that clinicians observe, and a diagnosis of chronic PVT is usually made by chance [14]. In a patient with an acquired hypercoagulable or primary pro-thrombotic state, chronic PVT can develop into acute PVT because of precipitating factors including pro-coagulants administered to treat bleeding. Acute PVT also has no specific symptoms and signs; intestinal ischemia and necrosis are typical clinical presentations, while abdominal pain, distention, diarrhea, and bleeding are manifestations. However, PVT it is usually not diagnosed at the early stage or before surgical interventions [15]. For our patient, PVT was diagnosed during laparotomy, not pre-transplant. If the thrombi do not resolve, ischemia and gut necrosis will eventually ensue, resulting in peritonitis and septic shock, which will preclude more complex liver transplantation. Patients with cirrhosis and high-grade PVT were shown to have low survival compared with the patients who were PVT-free (5), and their PVT recurrence rate is unacceptably high [16]. An explanation for why few PVT cases have been reported in LT candidates with acute PVT may be because perforation will probably lead to bacterial translocation, and peritonitis or septic shock will ensue, which will endanger the liver allograft and recipient’s life. The risk of recurrence and the mortality rate both remain high after LT for PVT. Additionally, an organ donor is not always available, or other patients on the waitlist are considered to be a higher priority to receive donor organs.

Gastrointestinal bleeding is a common complication of PVT. There is consensus that endoscopic variceal obturation is the first-line treatment option, and that it is beneficial to use anticoagulant drugs if the thrombus extends into the PVT and superior mesenteric vein, even in the event of upper gastrointestinal bleeding when the diagnosis was made. Anticoagulant use is associated with low recurrence and high recanalization [17]. For this patient, thrombi seemed to be diffuse, and thrombectomy was used efficiently to ensure blood flow to the liver graft. This is a simple and useful procedure is usually the first choice, especially for acute thrombi. In the hypercoagulable state, thrombi form in the small vessels arising from the superior mesenteric vein, and predisposing factors involve the large veins. Thrombogenesis initiates compression at the site of the main portal vein, and it then extends distally to the superior mesenteric vein [18]. Thrombi that formed in the short time when they were loosely and sparsely adhered to the venous tunica intima were flushed by blood outflow in our patient. Michael et al. reported that phlebothrombectomy was performed successfully in 90 % of these patients [5]. To our knowledge, this is the first report that details treatment of an LT candidate with PVT followed by acute onset and subsequent intestinal ischemia.

## Conclusions

In conclusion, isolated resection of intestinal gangrene without perforation that results from acute PVT may not jeopardize liver transplantation in patients with end-stage cirrhosis. Multidisciplinary collaborations and improved imaging technology are needed to screen and prevent rapid progression of PVT at the early stage, and decisive and aggressive approaches are crucial for favorable outcomes.

## Abbreviations

LT: Liver transplantation
PVT: Portal vein thrombosis
